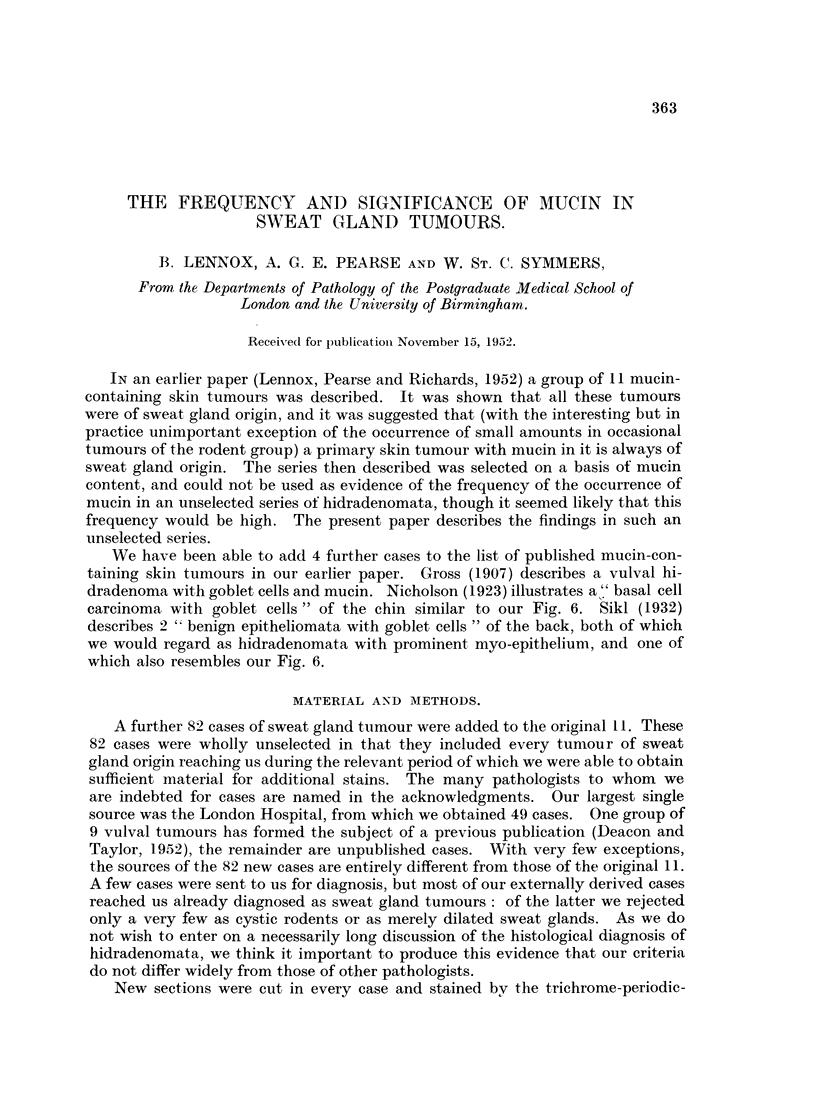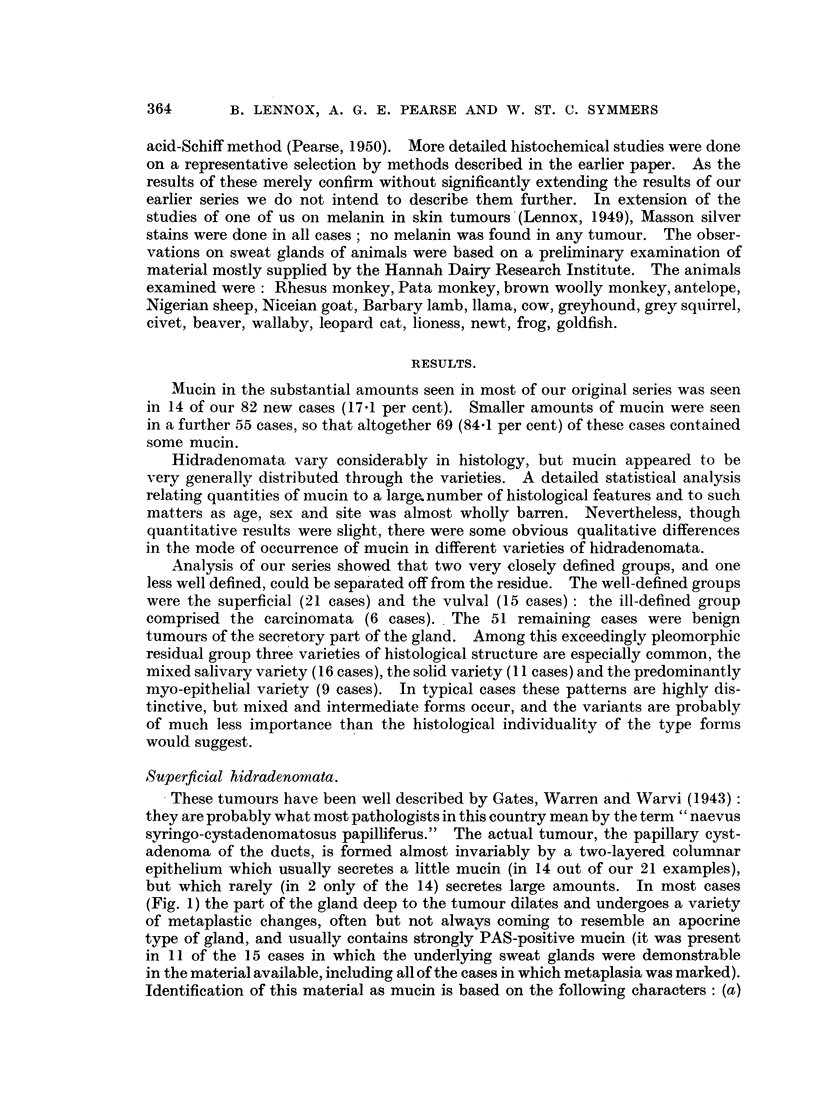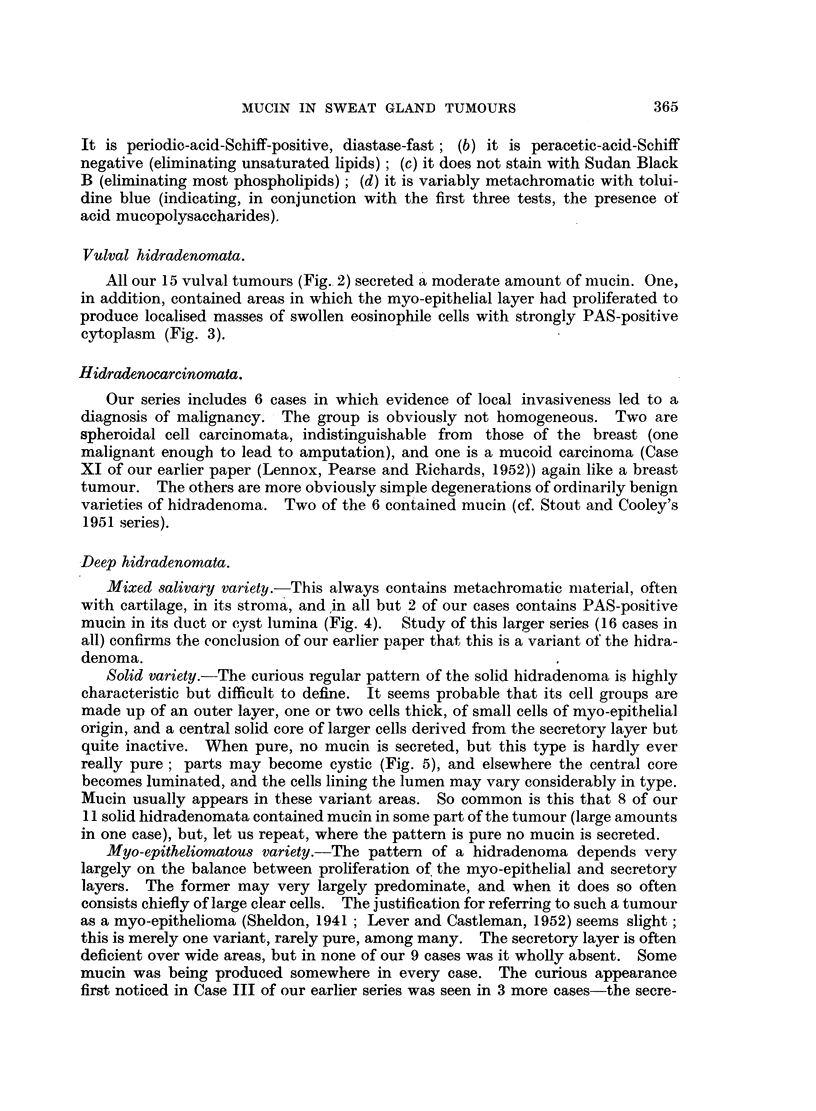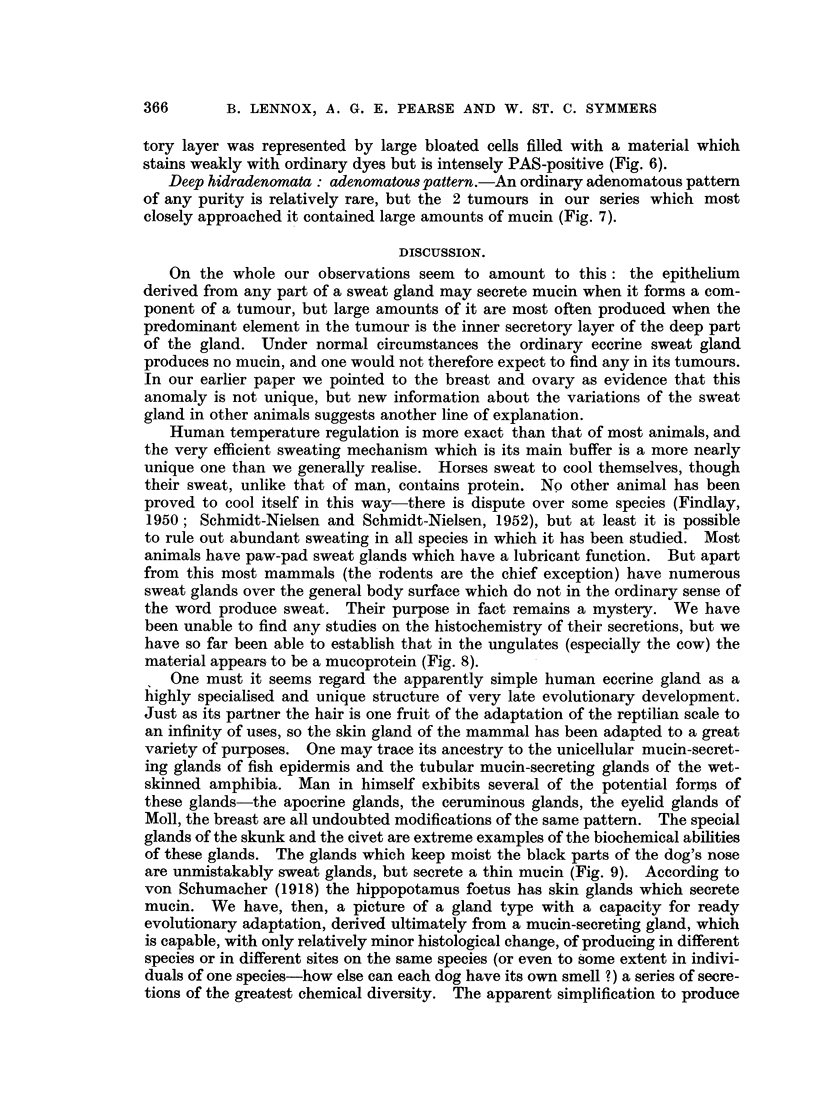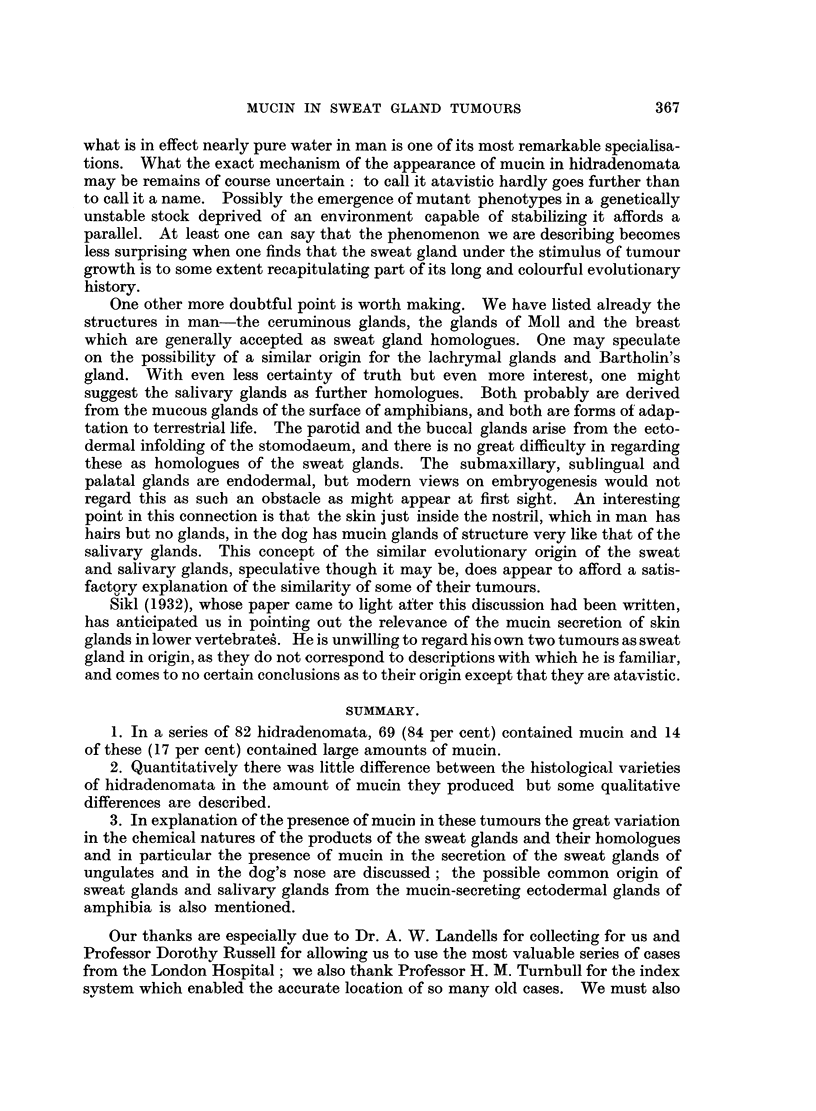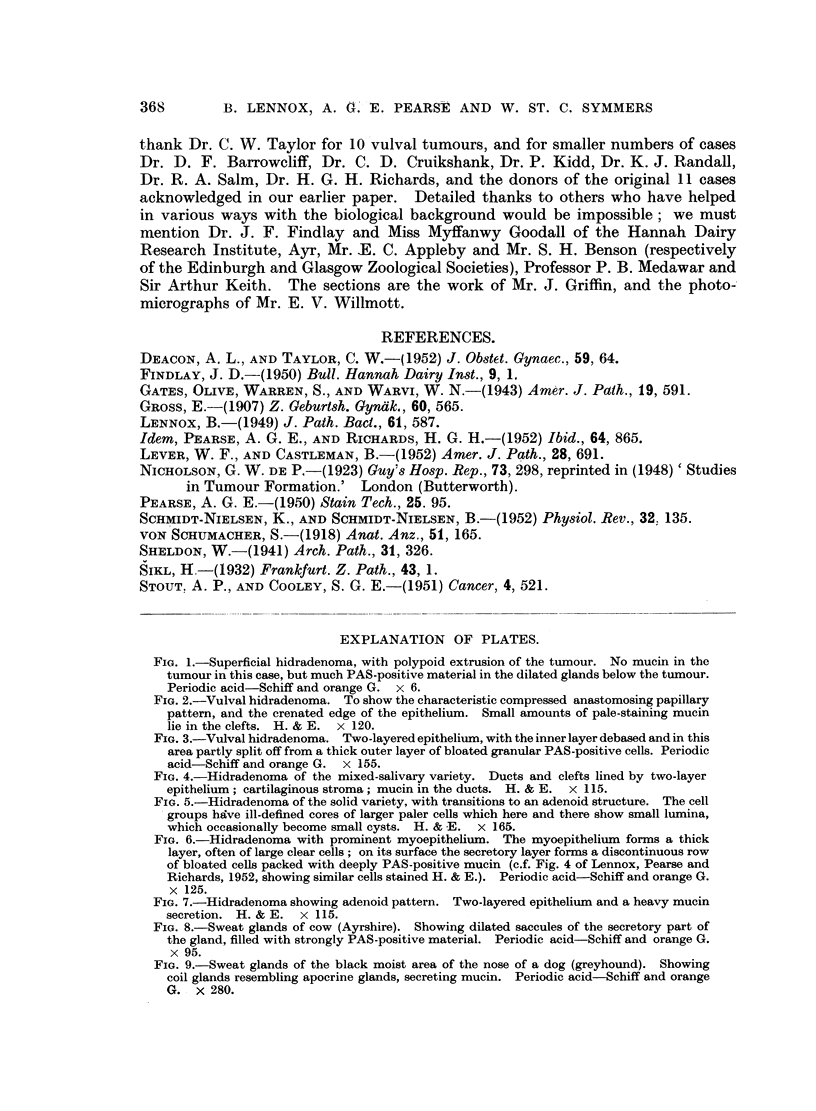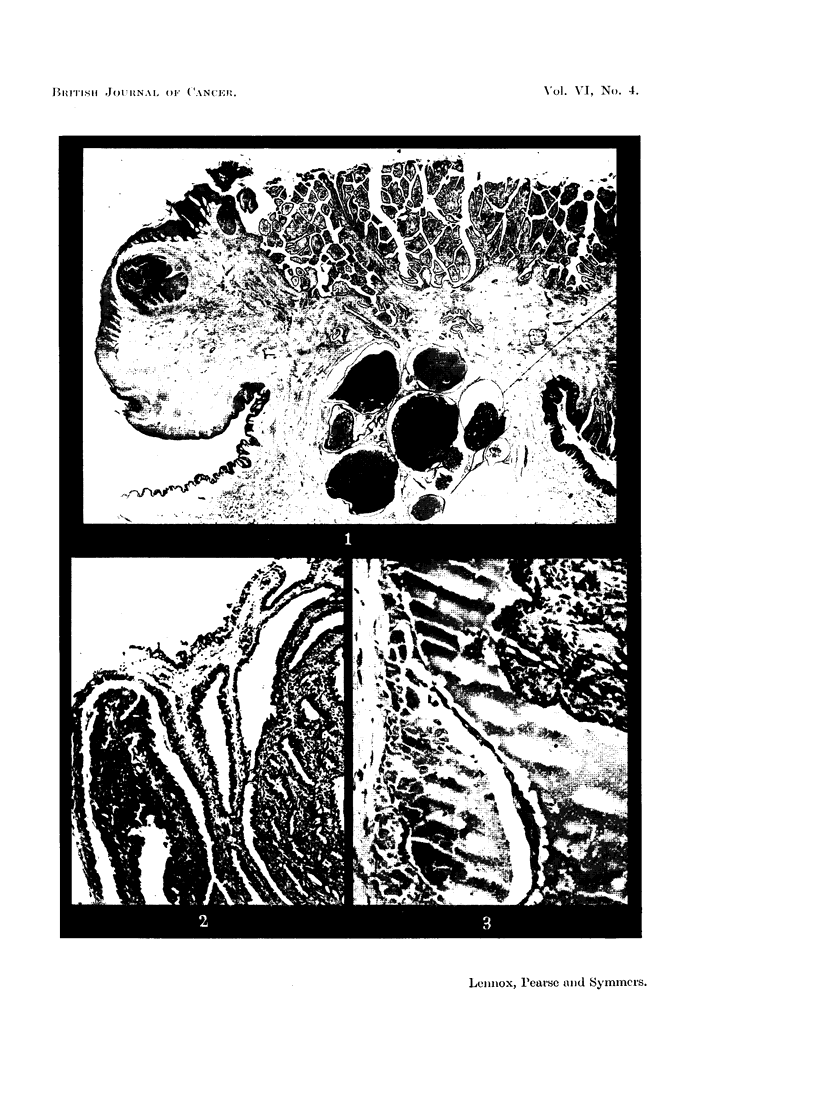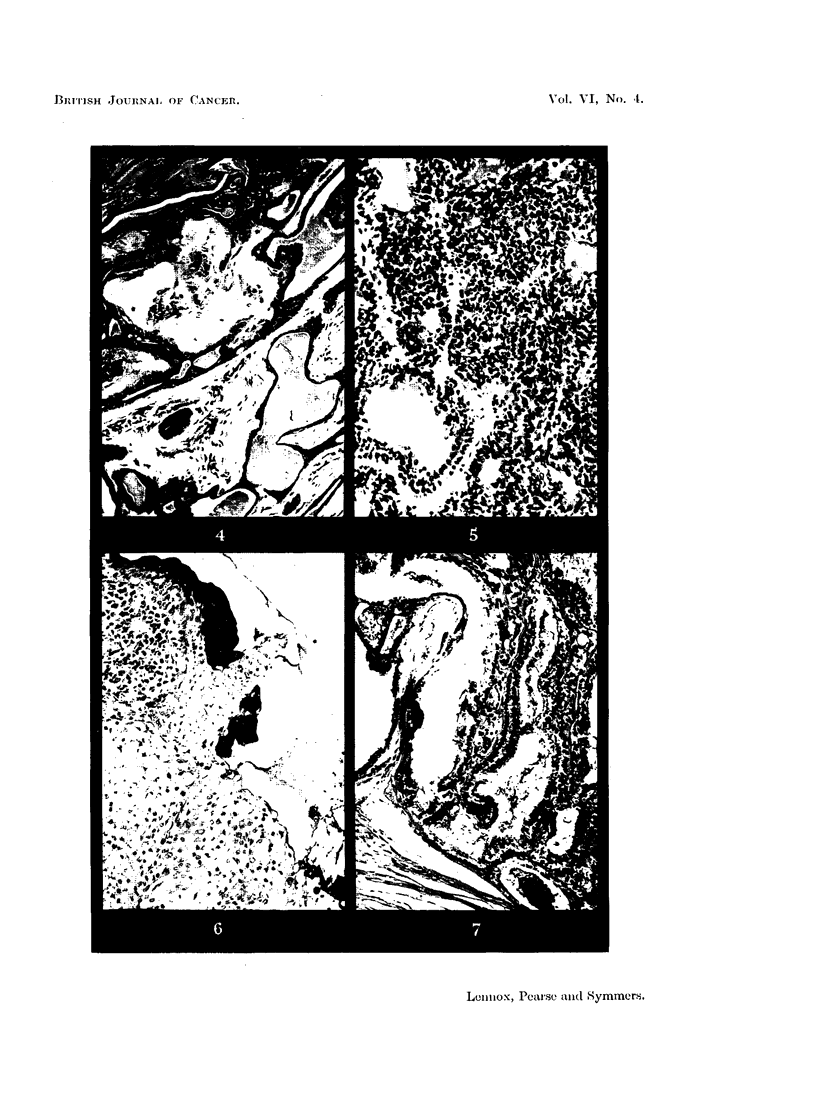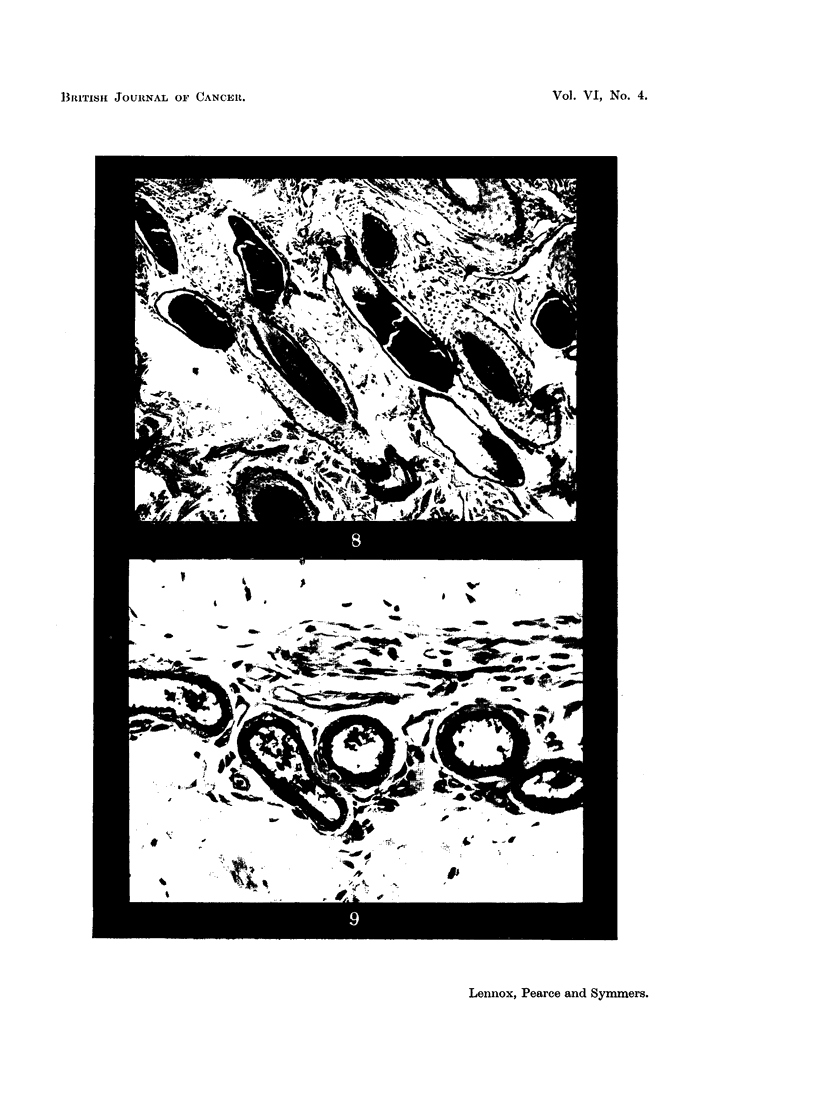# The Frequency and Significance of Mucin in Sweat Gland Tumours

**DOI:** 10.1038/bjc.1952.40

**Published:** 1952-12

**Authors:** B. Lennox, A. G. E. Pearse, W. St. C. Symmers

## Abstract

**Images:**


### THE FREQUENCY AND SIGNIFICANCE OF MUCIN IN SWEAT GLAND TUMOURS.

B. LENNOX, A. G. E. PEARSE AND W. ST. C. SYMMERS,

*From the Departments of Pathology of the Postgraduate Medical School of London and the University of Birmingham.*

Received for publication November 15, 1952.

IN an earlier paper (Lennox, Pearse and Richards, 1952) a group of 11 mucin-containing skin tumours was described. It was shown that all these tumours were of sweat gland origin, and it was suggested that (with the interesting but in practice unimportant exception of the occurrence of small amounts in occasional tumours of the rodent group) a primary skin tumour with mucin in it is always of sweat gland origin. The series then described was selected on a basis of mucin content, and could not be used as evidence of the frequency of the occurrence of mucin in an unselected series of hidradenomata, though it seemed likely that this frequency would be high. The present paper describes the findings in such an unselected series.

We have been able to add 4 further cases to the list of published mucin-containing skin tumours in our earlier paper. Gross (1907) describes a vulval hidradenoma with goblet cells and mucin. Nicholson (1923) illustrates a " basal cell carcinoma with goblet cells " of the chin similar to our Fig. 6. Sikl (1932) describes 2 " benign epitheliomata with goblet cells " of the back, both of which we would regard as hidradenomata with prominent myo-epithelium, and one of which also resembles our Fig. 6.

### MATERIAL AND METHODS.

A further 82 cases of sweat gland tumour were added to the original 11. These 82 cases were wholly unselected in that they included every tumour of sweat gland origin reaching us during the relevant period of which we were able to obtain sufficient material for additional stains. The many pathologists to whom we are indebted for cases are named in the acknowledgments. Our largest single source was the London Hospital, from which we obtained 49 cases. One group of 9 vulval tumours has formed the subject of a previous publication (Deacon and Taylor, 1952), the remainder are unpublished cases. With very few exceptions, the sources of the 82 new cases are entirely different from those of the original 11. A few cases were sent to us for diagnosis, but most of our externally derived cases reached us already diagnosed as sweat gland tumours : of the latter we rejected only a very few as cystic rodents or as merely dilated sweat glands. As we do not wish to enter on a necessarily long discussion of the histological diagnosis of hidradenomata, we think it important to produce this evidence that our criteria do not differ widely from those of other pathologists.

New sections were cut in every case and stained by the trichrome-periodicacid-Schiff method (Pearse, 1950). More detailed histochemical studies were done on a representative selection by methods described in the earlier paper. As the results of these merely confirm without significantly extending the results of our earlier series we do not intend to describe them further. In extension of the studies of one of us on melanin in skin tumours (Lennox, 1949), Masson silver stains were done in all cases ; no melanin was found in any tumour. The observations on sweat glands of animals were based on a preliminary examination of material mostly supplied by the Hannah Dairy Research Institute. The animals examined were : Rhesus monkey, Pata monkey, brown woolly monkey, antelope, Nigerian sheep, Niceian goat, Barbary lamb, llama, cow, greyhound, grey squirrel, civet, beaver, wallaby, leopard cat, lioness, newt, frog, goldfish.

### RESULTS.

Mucin in the substantial amounts seen in most of our original series was seen in 14 of our 82 new cases (17·1 per cent). Smaller amounts of mucin were seen in a further 55 cases, so that altogether 69 (84·1 per cent) of these cases contained some mucin.

Hidradenomata vary considerably in histology, but mucin appeared to be very generally distributed through the varieties. A detailed statistical analysis relating quantities of mucin to a large number of histological features and to such matters as age, sex and site was almost wholly barren. Nevertheless, though quantitative results were slight, there were some obvious qualitative differences in the mode of occurrence of mucin in different varieties of hidradenomata.

Analysis of our series showed that two very closely defined groups, and one less well defined, could be separated off from the residue. The well-defined groups were the superficial (21 cases) and the vulval (15 cases) : the ill-defined group comprised the carcinomata (6 cases). The 51 remaining cases were benign tumours of the secretory part of the gland. Among this exceedingly pleomorphic residual group three varieties of histological structure are especially common, the mixed salivary variety (16 cases), the solid variety (11 cases) and the predominantly myo-epithelial variety (9 cases). In typical cases these patterns are highly distinctive, but mixed and intermediate forms occur, and the variants are probably of much less importance than the histological individuality of the type forms would suggest.

### *Superficial hidradenomata.*

These tumours have been well described by Gates, Warren and Warvi (1943) : they are probably what most pathologists in this country mean by the term "naevus syringo-cystadenomatosus papilliferus." The actual tumour, the papillary cystadenoma of the ducts, is formed almost invariably by a two-layered columnar epithelium which usually secretes a little mucin (in 14 out of our 21 examples), but which rarely (in 2 only of the 14) secretes large amounts. In most cases (Fig. 1) the part of the gland deep to the tumour dilates and undergoes a variety of metaplastic changes, often but not always coming to resemble an apocrine type of gland, and usually contains strongly PAS-positive mucin (it was present in 11 of the 15 cases in which the underlying sweat glands were demonstrable in the material available, including all of the cases in which metaplasia was marked). Identification of this material as mucin is based on the following characters : (*a*)

It is periodic-acid-Schiff-positive, diastase-fast ; (*b*) it is peracetic-acid-Schiff negative (eliminating unsaturated lipids) ; (*c*) it does not stain with Sudan Black B (eliminating most phospholipids) ; (*d*) it is variably metachromatic with toluidine blue (indicating, in conjunction with the first three tests, the presence of acid mucopolysaccharides).

*Vulval hidradenomata.*

All our 15 vulval tumours (Fig. 2) secreted a moderate amount of mucin. One, in addition, contained areas in which the myo-epithelial layer had proliferated to produce localised masses of swollen eosinophile cells with strongly PAS-positive cytoplasm (Fig. 3).

*Hidradenocarcinomata.*

Our series includes 6 cases in which evidence of local invasiveness led to a diagnosis of malignancy. The group is obviously not homogeneous. Two are spheroidal cell carcinomata, indistinguishable from those of the breast (one malignant enough to lead to amputation), and one is a mucoid carcinoma (Case XI of our earlier paper (Lennox, Pearse and Richards, 1952)) again like a breast tumour. The others are more obviously simple degenerations of ordinarily benign varieties of hidradenoma. Two of the 6 contained mucin (cf. Stout and Cooley's 1951 series).

*Deep hidradenomata.*

*Mixed salivary variety.*—This always contains metachromatic material, often with cartilage, in its stroma, and in all but 2 of our cases contains PAS-positive mucin in its duct or cyst lumina (Fig. 4). Study of this larger series (16 cases in all) confirms the conclusion of our earlier paper that this is a variant of the hidradenoma.

*Solid variety.*—The curious regular pattern of the solid hidradenoma is highly characteristic but difficult to define. It seems probable that its cell groups are made up of an outer layer, one or two cells thick, of small cells of myo-epithelial origin, and a central solid core of larger cells derived from the secretory layer but quite inactive. When pure, no mucin is secreted, but this type is hardly ever really pure ; parts may become cystic (Fig. 5), and elsewhere the central core becomes luminated, and the cells lining the lumen may vary considerably in type. Mucin usually appears in these variant areas. So common is this that 8 of our 11 solid hidradenomata contained mucin in some part of the tumour (large amounts in one case), but, let us repeat, where the pattern is pure no mucin is secreted.

*Myo-epitheliomatous variety.*—The pattern of a hidradenoma depends very largely on the balance between proliferation of the myo-epithelial and secretory layers. The former may very largely predominate, and when it does so often consists chiefly of large clear cells. The justification for referring to such a tumour as a myo-epithelioma (Sheldon, 1941 ; Lever and Castleman, 1952) seems slight ; this is merely one variant, rarely pure, among many. The secretory layer is often deficient over wide areas, but in none of our 9 cases was it wholly absent. Some mucin was being produced somewhere in every case. The curious appearance first noticed in Case III of our earlier series was seen in 3 more cases—the secretory layer was represented by large bloated cells filled with a material which stains weakly with ordinary dyes but is intensely PAS-positive (Fig. 6).

*Deep hidradenomata : adenomatous pattern.*—An ordinary adenomatous pattern of any purity is relatively rare, but the 2 tumours in our series which most closely approached it contained large amounts of mucin (Fig. 7).

### DISCUSSION.

On the whole our observations seem to amount to this : the epithelium derived from any part of a sweat gland may secrete mucin when it forms a component of a tumour, but large amounts of it are most often produced when the predominant element in the tumour is the inner secretory layer of the deep part of the gland. Under normal circumstances the ordinary eccrine sweat gland produces no mucin, and one would not therefore expect to find any in its tumours. In our earlier paper we pointed to the breast and ovary as evidence that this anomaly is not unique, but new information about the variations of the sweat gland in other animals suggests another line of explanation.

Human temperature regulation is more exact than that of most animals, and the very efficient sweating mechanism which is its main buffer is a more nearly unique one than we generally realise. Horses sweat to cool themselves, though their sweat, unlike that of man, contains protein. No other animal has been proved to cool itself in this way—there is dispute over some species (Findlay, 1950 ; Schmidt-Nielsen and Schmidt-Nielsen, 1952), but at least it is possible to rule out abundant sweating in all species in which it has been studied. Most animals have paw-pad sweat glands which have a lubricant function. But apart from this most mammals (the rodents are the chief exception) have numerous sweat glands over the general body surface which do not in the ordinary sense of the word produce sweat. Their purpose in fact remains a mystery. We have been unable to find any studies on the histochemistry of their secretions, but we have so far been able to establish that in the ungulates (especially the cow) the material appears to be a mucoprotein (Fig. 8).

One must it seems regard the apparently simple human eccrine gland as a highly specialised and unique structure of very late evolutionary development. Just as its partner the hair is one fruit of the adaptation of the reptilian scale to an infinity of uses, so the skin gland of the mammal has been adapted to a great variety of purposes. One may trace its ancestry to the unicellular mucin-secreting glands of fish epidermis and the tubular mucin-secreting glands of the wet-skinned amphibia. Man in himself exhibits several of the potential forms of these glands—the apocrine glands, the ceruminous glands, the eyelid glands of Moll, the breast are all undoubted modifications of the same pattern. The special glands of the skunk and the civet are extreme examples of the biochemical abilities of these glands. The glands which keep moist the black parts of the dog's nose are unmistakably sweat glands, but secrete a thin mucin (Fig. 9). According to von Schumacher (1918) the hippopotamus foetus has skin glands which secrete mucin. We have, then, a picture of a gland type with a capacity for ready evolutionary adaptation, derived ultimately from a mucin-secreting gland, which is capable, with only relatively minor histological change, of producing in different species or in different sites on the same species (or even to some extent in individuals of one species—how else can each dog have its own smell ?) a series of secretions of the greatest chemical diversity. The apparent simplification to produce what is in effect nearly pure water in man is one of its most remarkable specialisations. What the exact mechanism of the appearance of mucin in hidradenomata may be remains of course uncertain : to call it atavistic hardly goes further than to call it a name. Possibly the emergence of mutant phenotypes in a genetically unstable stock deprived of an environment capable of stabilizing it affords a parallel. At least one can say that the phenomenon we are describing becomes less surprising when one finds that the sweat gland under the stimulus of tumour growth is to some extent recapitulating part of its long and colourful evolutionary history.

One other more doubtful point is worth making. We have listed already the structures in man—the ceruminous glands, the glands of Moll and the breast which are generally accepted as sweat gland homologues. One may speculate on the possibility of a similar origin for the lachrymal glands and Bartholin's gland. With even less certainty of truth but even more interest, one might suggest the salivary glands as further homologues. Both probably are derived from the mucous glands of the surface of amphibians, and both are forms of adaptation to terrestrial life. The parotid and the buccal glands arise from the ectodermal infolding of the stomodaeum, and there is no great difficulty in regarding these as homologues of the sweat glands. The submaxillary, sublingual and palatal glands are endodermal, but modern views on embryogenesis would not regard this as such an obstacle as might appear at first sight. An interesting point in this connection is that the skin just inside the nostril, which in man has hairs but no glands, in the dog has mucin glands of structure very like that of the salivary glands. This concept of the similar evolutionary origin of the sweat and salivary glands, speculative though it may be, does appear to afford a satisfactory explanation of the similarity of some of their tumours.

Sikl (1932), whose paper came to light after this discussion had been written, has anticipated us in pointing out the relevance of the mucin secretion of skin glands in lower vertebrates. He is unwilling to regard his own two tumours as sweat gland in origin, as they do not correspond to descriptions with which he is familiar, and comes to no certain conclusions as to their origin except that they are atavistic.

### SUMMARY.

1. In a series of 82 hidradenomata, 69 (84 per cent) contained mucin and 14 of these (17 per cent) contained large amounts of mucin.

2. Quantitatively there was little difference between the histological varieties of hidradenomata in the amount of mucin they produced but some qualitative differences are described.

3. In explanation of the presence of mucin in these tumours the great variation in the chemical natures of the products of the sweat glands and their homologues and in particular the presence of mucin in the secretion of the sweat glands of ungulates and in the dog's nose are discussed ; the possible common origin of sweat glands and salivary glands from the mucin-secreting ectodermal glands of amphibia is also mentioned.

Our thanks are especially due to Dr. A. W. Landells for collecting for us and Professor Dorothy Russell for allowing us to use the most valuable series of cases from the London Hospital ; we also thank Professor H. M. Turnbull for the index system which enabled the accurate location of so many old cases. We must also thank Dr. C. W. Taylor for 10 vulval tumours, and for smaller numbers of cases Dr. D. F. Barrowcliff, Dr. C. D. Cruikshank, Dr. P. Kidd, Dr. K. J. Randall, Dr. R. A. Salm, Dr. H. G. H. Richards, and the donors of the original 11 cases acknowledged in our earlier paper. Detailed thanks to others who have helped in various ways with the biological background would be impossible ; we must mention Dr. J. F. Findlay and Miss Myffanwy Goodall of the Hannah Dairy Research Institute, Ayr, Mr. E. C. Appleby and Mr. S. H. Benson (respectively of the Edinburgh and Glasgow Zoological Societies), Professor P. B. Medawar and Sir Arthur Keith. The sections are the work of Mr. J. Griffin, and the photomicrographs of Mr. E. V. Willmott.

### REFERENCES.

Deacon, A. L., and Taylor, C. W.—(1952) *J. Obstet. Gynaec.*, **59**, 64.
Findlay, J. D.—(1950) *Bull. Hannah Dairy Inst.*, **9**, 1.
Gates, Olive, Warren, S., and Warvi, W. N.—(1943) *Amer. J. Path.*, **19**, 591.
Gross, E.—(1907) *Z. Geburtsh. Gynäk.*, **60**, 565.
Lennox, B.—(1949) *J. Path. Bact.*, **61**, 587.
*Idem*, Pearse, A. G. E., and Richards, H. G. H.—(1952) *Ibid.*, **64**, 865.
Lever, W. F., and Castleman, B.—(1952) *Amer. J. Path.*, **28**, 691.
Nicholson, G. W. de P.—(1923) *Guy's Hosp. Rep.*, **73**, 298, reprinted in (1948) ' Studies in Tumour Formation.' London (Butterworth).
Pearse, A. G. E.—(1950) *Stain Tech.*, **25**. 95.
Schmidt-Nielsen, K., and Schmidt-Nielsen, B.—(1952) *Physiol. Rev.*, **32**, 135.
von Schumacher, S.—(1918) *Anat. Anz.*, **51**, 165.
Sheldon, W.—(1941) *Arch. Path.*, **31**, 326.
Šikl, H.—(1932) *Frankfurt. Z. Path.*, **43**, 1.
Stout, A. P., and Cooley, S. G. E.—(1951) *Cancer*, **4**, 521.

---

### EXPLANATION OF PLATES.

Fig. 1.—Superficial hidradenoma, with polypoid extrusion of the tumour. No mucin in the tumour in this case, but much PAS-positive material in the dilated glands below the tumour. Periodic acid—Schiff and orange G. × 6.

Fig. 2.—Vulval hidradenoma. To show the characteristic compressed anastomosing papillary pattern, and the crenated edge of the epithelium. Small amounts of pale-staining mucin lie in the clefts. H. & E. × 120.

Fig. 3.—Vulval hidradenoma. Two-layered epithelium, with the inner layer debased and in this area partly split off from a thick outer layer of bloated granular PAS-positive cells. Periodic acid—Schiff and orange G. × 155.

Fig. 4.—Hidradenoma of the mixed-salivary variety. Ducts and clefts lined by two-layer epithelium ; cartilaginous stroma ; mucin in the ducts. H. & E. × 115.

Fig. 5.—Hidradenoma of the solid variety, with transitions to an adenoid structure. The cell groups have ill-defined cores of larger paler cells which here and there show small lumina, which occasionally become small cysts. H. & E. × 165.

Fig. 6.—Hidradenoma with prominent myoepithelium. The myoepithelium forms a thick layer, often of large clear cells ; on its surface the secretory layer forms a discontinuous row of bloated cells packed with deeply PAS-positive mucin (c.f. Fig. 4 of Lennox, Pearse and Richards, 1952, showing similar cells stained H. & E.). Periodic acid—Schiff and orange G. × 125.

Fig. 7.—Hidradenoma showing adenoid pattern. Two-layered epithelium and a heavy mucin secretion. H. & E. × 115.

Fig. 8.—Sweat glands of cow (Ayrshire). Showing dilated saccules of the secretory part of the gland, filled with strongly PAS-positive material. Periodic acid—Schiff and orange G. × 95.

Fig. 9.—Sweat glands of the black moist area of the nose of a dog (greyhound). Showing coil glands resembling apocrine glands, secreting mucin. Periodic acid—Schiff and orange G. × 280.